# Historical Sketch of the Progress of Medicine in the Year 1808

**Published:** 1809-07

**Authors:** 


### Medical and Physical Journal.

VOL. XXII.] JULY, 1809. [NO. 125.

*Printed for R. PHILLIPS, by W. Thorne, Red Lion Court, Fleet Street, London.*

### HISTORICAL SKETCH OF THE PROGRESS OF MEDICINE IN THE YEAR 1808; BY MR. ROYSTON.

### No. 3.

( To be continued Annually. )

*Grateful, as I am, to the good Being whose bounty has imparted to me this reasoning intellect, whatever it is, I hold myself proportionably indebted to him, from whose enlightened understanding another ray of knowledge communicates to mine.*

JUNIUS.

IF it were possible to trace the progress of science from the earliest times, with the same precision that history has followed the footsteps of the conqueror, or unfolded the causes of national revolutions; a book might be formed that would have strong claims to the attention of mankind. Could the veil be withdrawn that covers, with perhaps designed obscurity, the arts of ancient Egypt; and the mysterious mythology be penetrated, that involves the literature even of Greece; it might become evident that the powers of human intellect had risen in these nations to a height that modern science would not disdain. And in the erudition of the antient world, whether of China, India, Egypt, or Greece, did some genius arise powerful enough to remove the clouds that interpose, possibly many of the discoveries of modern times might be found. Even the Celtic nations, mingled with their wild and awful superstitions, a wonderful knowldge of mechanical powers, or of chemical operations.

There is, however, one department of human knowledge in which the ancients were remarkably defective, if the records of their attainments have not perished, or remain yet concealed in the darkness of symbolical representation: With very few exceptions, the operations of the vital energies and the laws of nature, seem to have been neglected, or strangely misunderstood, by the sages of antiquity. What is called experimental philosophy is an invention of modern times; and medicine, as a science founded on incontrovertible facts, and a true observance of nature, existed in a state of extreme imperfection till long after the revival of literature; though it might be proved that the physicians of Greece and Rome possessed an extraordinary dexterity in some practical points, intimately combined with a disposition to form groundless theories, and hypothetical systems.

Notwithstanding the obscurity on one hand, and the puerile and fanciful inventions on the other, enough of certainty remains to make it felt, in contemplating the progress that mankind has made toward a knowledge of many arts, as well as the melioration of intellect on subjects peculiarly applicable to mind, that the materials upon which this mechanic dexterity or mental illumination are founded, have been accumulating from age to age; and also that the skill of various periods has, from want of sufficient records, been sometimes entirely lost, and at others imperfectly transmitted. It will too be obvious, that had authentic memorials of knowledge descended with the progress of time, and bequeathed fully the stream of intellect, unobstructed and unimpaired, from generation to generation, that an acmé in science had been attained, of which, perhaps, we have now but a faint image. This defect has been removed by the discoveries of modern times. The learning of distant regions, as it might have been of remote ages, has been made to approximate; and the aggregate of knowledge, like some mighty engine, bears down the barriers that seem for ages to have said, thus far shalt thou go and no farther, and here shall thy inquiries be stayed.

With the advantage of difficulties, once deemed insuperable, surmounted; enlightened by discoveries of which former ages had not, as far as is known, the slightest intimation; it must be expected that an acquaintance with the operations of Nature is in a state of progression. And notwithstanding much of the knowledge of the early ages of the world may be lost from the want of means that would have multiplied the evidence of its existence, it will be admitted that advances are daily making toward a developement of the whole system of Nature, that promise, at no very distant period, to explain the principles of of animation, and ascertain, more *fully*, the laws that govern the actions of vitality.

To methodize, abridge, and occasionally explain this progress of science, has been deemed an advantageous mode of promoting general knowledge; and conducive particularly to the improvement of the practical part of medicine, as rationally founded on an acquaintance with the structure and functions of animal bodies, and extensive views of the laws that govern and direct the operations in the systems of all creatures possessing life.

In a comprehensive designation of the Medical Art, Natural History, Botany, Chemistry, and Experimental Philosophy in many, if not all its branches, as well as Anatomy and Physiology, will be but elementary parts. And a persuasion that the more distinct is the knowledge of these elementary parts possessed by the medical practitioner, the more perfectly will he comprehend morbid phenomena, and feel the duties and claims of an arduous employment, has occasioned some preliminary notice to be taken of them, as subservient to a view of the practical part of medicine.

ANATOMY and Physiology, or a knowledge of the structure and functions of animated Nature, is so essential a part of medical education, and the principles resulting from this knowledge are so fundamentally important, that no collateral acquirements, however brilliant or extensive, can compensate for their absence. To recommend a sedulous application to this branch of elementary knowledge would be superfluous, if in a well written essay in a respectable periodical publication, an endeavour had not been made to seduce the student from this essential object, by a chain of ingenious and plausible arguments. An assumption that a minute knowledge of anatomy leads to a conclusion that the animal body is a mere machine, without any reference to the operations of the principle of vitality, were it true, would be a decisive argument. If in the days of Borelli and Keil this notion of animal mechanism prevailed, the converse is the fact at present; and inquiry is now probably too much employed on the inscrutable essence of the principle of vitality: its operations on the functions, and its controul over animal organization, being used in a vague manner to explain the nature of morbid actions, and the modus operandi of medicines. It would be fortunate if the discoverers of remedies, or of scientific principles, were always or often able to retrace their knowledge to the obscure hints and lucky circumstances, from whence they are asserted to be commonly derived. But the truth is, that the principles of science, and the efficient practice of arts, have not arisen out of accidents that give no more than loose hints; but are the result of study and reflection, in minds informed and enlarged by an acquaintance with elementary principles, and extensive views of Nature. Certainly both the materia medica and the practice of physic have sometimes been indebted to accident for medicinal substances, and their application in diseases; but the judicious employment of remedies has arisen out of an extensive acquaintance with the structure and functions of animals, connected with a knowledge of the properties of the substance employed, the nature and course of morbid derangements, and a nice adjustment of these with the age, temperament, and idiosyncracy of the patient. Though anatomy has not explained the properties and powers of remedies, without a reference to their effects on the living body;—though it has not taught, abstractedly, that cinchona is a bitter and a tonic, that opium is soporific or stimulant, that mercury cures syphilis, and that antimony is a powerful evacuant; yet it has shewn, by explaining the operations of the animal frame, how to regulate the actions of these powerful substances, and to perceive more distinctly the effects they produce on various parts of the system. It will be admitted, without reluctance, that mere anatomical knowledge does not make the practitioner; but yet is it an essential elementary part of that character. Conjoined with chemistry, botany, a knowledge of the materia medica, and the study of the symptoms of disease, anatomy forms the accomplished physician; and, perhaps, it gives the foundation and base of a pyramid, the apex of which terminates in rational practice. Whoever is an anatomist, may make a useful, a rational, and an excellent practitioner:—but he who is ignorant in this essential point, whatever are his other acquirements, will often be confused, irresolute, and hesitating, when he should be prompt and decisive.

Notwithstanding the evident value of this branch of elementary knowledge, the year 1808 has neither been fruitful of anatomical and physiological discovery, or of works, that from this source elucidate the practice of physic and surgery.

A subject of much intricacy even in the apparent and common actions of the frame, and of profound obscurity as to the cause of these actions, has been investigated by

Dr.

Dr. Barclay, in a work on the muscular motions of the human body. The difficulty of treating an abstruse subject with clearness and precision, must always be increased by the compulsory use of a language to which the reader is unaccustomed, and of terms that in every page require a definition. But surely the choice of such a language and of such terms, if a writer means to be understood, is most unfortunate. A few years since Dr. Barclay published an anatomical nomenclature, the terms of which have not become current, but they are adopted into this work. And though the employment of a language peculiar to the author, and which few beside himself will understand, has not entirely suppressed the interest, it has spread an obscurity and gloom over a work calculated to claim the attention of physiologists, by giving a comprehensive view of the structure of the muscular fibre, the nature and laws of irritability, and the simple and combined actions of muscles. If the descriptions, from the singularity of the language in which they are given, want that graphic determination that brings the object before the eye; * if the observations and the opinions are sometimes problematical from a redundance of scientific refinement; the student, and perhaps the practitioner, may be rewarded with useful information by the perusal of a work, which they must labour to understand.

The extended and general views of Dr. Barclay, are contrasted by the particular application of the ingenuity of Mr. Home in descriptions of the structure, and in illustration of the functions of some individual viscus. In a series of experiments and observations on the functions of the stomach, printed in the Phil. Trans. for 1807, and noticed in this Report for that year, Mr. Home believed he had ascertained, that while digestion is going on, there is a separation between the cardiac and pyloric portions, either by means of a permanent or muscular contraction; and that this fact placed the process of digestion in a new light, and led him to consider in what way the quantities of different liquors, which are so often taken into the stomach, can be prevented from mixing with the half digest-

* The motions of the scapula, as described by Dr. B. are atlantad, sacrad, ster-mesilaterad, dorsa-mesial, rotatory, or combinations of these motions. In moving sternad, it cannot reach the mesial plane; on account of the clavicle, it must follow a direction that is ster-mesi-lateral, and when its basis approaches that plane on the dorsal aspect, compelled by the ribs, it must move in a plane that is dorsa-mesial, &c. &c. &c.

ed food, and interfering with the formation of the chyle. Pursuing this inquiry, he found that the fluids are princit pally contained in the cardiac portion, and the food tha has reached the pyloric portion is usually of one uniform consistence, so that the fluids, beyond what are necessary for digestion, would appear to be carried out of the stomach, without ever reaching so far as the pylorus. But out of this new light in the process of digestion, a question arose; it was, *how did these superabundant fluids escape from the stomach?* Many circumstances, said Mr. Home, appeared to render it probable that the *Spleen* was the route by which they were conveyed; and this opinion gave rise to two papers in the Phil. Trans. for this year, on the structure and use of that viscus.

In these papers, Mr. Home endeavours to prove by experiment, the truth of a position at first hypothetical. A concurrence of favourable circumstances rendered the Spleen a commodious resort. Its vicinity to the stomach, its texture, and above all its unclaimed services in the animal œconomy, point it out for employment on a variety of occasions. To ascertain, among its other destinations, that it is intended to serve as a reservoir for receiving the superfluous fluid that is taken into the stomach, Mr. Home, or his assistants, put a ligature about the pylorus, and injected a quantity of fluid into the stomach. After some time, part of the fluid had disappeared; there was no visible trace of it in the absorbent vessels; but the Spleen was become turgid, and its cells were found distended with a fluid. To identify this fluid found in the Spleen with the portion remaining in the stomach, an infusion of rhubarb was given to an animal, and after some time, the colouring matter was detected in the cells of the Spleen, though it could not be perceived in other parts of the body. By experiments on the ass, it was also ascertained that a fluid coloured with rhubarb passed from the stomach to the Spleen, when the pylorus was left open. The experiments contained in these papers Mr. Home believes have ascertained,

" That the spleen is met with in two very different states, one which may be termed the distended, the other the contracted; and that in one its size is double what it is in the other. In the distended state there is a distinct appearance of cells, containing a limpid fluid, distinguishable by the naked eye; in the contracted those only become distinct when seen through a magnifying glass. The distended state takes place when the stomach has received unusual unusual quantities of liquids before the animal's death; and the contracted state, when the animal has been kept several days without drink before the spleen is examined.

"That when the pylorus is secured, coloured fluids pass from the cardiac portion of the stomach into the circulation of the blood, and go off by urine; and while this is going on, the spleen is in its most distended state, and the colouring matter is found in its juices, although it is not to be detected in those of the liver. The colouring matter cannot therefore be conveyed to the spleen through the common absorbents of the stomach, which lead to the thoracic duct.

"That when the spleen is in this state, the blood in the splenic vein has its serum more strongly impregnated with the colouring matter, than the blood in the other veins of the body; and when the stomach is kept without liquids, although colouring matter is carried into the system from the intestinal canal by the ordinary channels, no particular evidence of it is met with in the spleen or its veins." The conclusions drawn from these statements are,

"That the liquids received into the stomach beyond what are employed for digestion, are not wholly carried out by the common absorbents of the stomach, or the canal of the intestines, *but are partly conveyed through the medium of the spleen into the circulation of the liver.*

"That if the vessels which communicate between the stomach and the spleen have not been discovered, the foregoing experiments are strongly presumptive of the existence of such vessels.

"That this communication between the cardiac portion of the stomach and the spleen, will explain the circumstances of those who are in the habit of drinking spirituous liquors having the spleen and liver so frequently diseased, and the diseases of both organs being of the same kind.

"That the considerable disease or removal of this organ, (though it is not essential to life, its office being of a secondary nature) must occasion digestion to be disturbed."

Well imagined and correctly performed experiments afford the only certain mode of arriving at truth in the operations of nature; and the uses of the organs of animals ascertained by this means, are established on a basis that philosophy admits to be indisputable. How far Mr. Home has succeeded in this instance will be left to future experimenters to determine; or of what advantage, if he has

 succeeded, succeeded, his discovery will be to pathology, is not very obvious. But let the final result be whatever it may, science is indebted to him for keeping in view this method of inquiry by experiment, and of making deductions from facts.

The direct progress of anatomy is not, perhaps, to be traced farther; but much collateral matter, connected more or less with this subject, has been given to the public. Under the disputed and disputable term morbid anatomy, no inconsiderable portion of talent has been exerted, as will hereafter be noticed; and the history of man generally, or particular modifications of his frame and diseases, have received some illustration from Dr. Jarrold and Dr. Reeve. The *Anthropologia* of the former, the professed object of which is to "remove every unwarranted prejudice against the person of the negro, and to call the attention of parents to those means by which the beauty and strength of their offspring may be improved," contains a series of dissertations on the natural history of man.

The art of medicine, taken in the extensive sense in which we have always been anxious to view it, will include a very large portion of those objects, in every class of nature, which the author of the universe has destined the intellect of man to investigate; and upon many of which, the volumes of this work are largely employed. The gradations of created beings, or the relations they bear to each other, their connections and dependencies, are circumstances of great interest to the medical philosopher; and if Dr. Jarrold's opposition to the opinions of Mr. White on this subject, do not carry with them a full conviction, they lead the mind on to further views and additional investigations. The effect of climate, of habit, of food, on the human frame, is an object of curious research, as explanatory of the external appearances of man. The hazardous question of the origin of the human race from one stock is discussed; and without going very deeply into the inquiry, it is determined that the races of man, however distinguished by peculiarities of form and colour, in the islands of the South Sea, in Lapland, the continent of America, Hindoostan, Tartary, and the polished nations of Europe, have the same individual origin. How these varieties were produced, is a discussion of deep interest to the philosopher; and to the Christian and the Deist, a question of great intricacy and difficult solution. Among the races of men, the most obvious distinguishing peculiarity is the colour of the skin, which varies through every shade from white white to black; and the causes of this peculiarity are inquired into at some length. The direct source of colour, or the colouring principle, is asserted to be iron; and in animals and vegetables, the presence of this mineral, under some modification, decides the tint. The presence of light or the sun's rays, are, however, a *sine qua non* of colour; and it is argued that the solar rays, with the assistance of moisture, which is considered as a *mordant*, produce the dark colour of the skin in man. Chemical science, however, will not explain this. That the vital principle is concerned in the production of colour seems very probable. In a living animal, with the aid of moisture, the solar rays deepen the colour; but deprive that animal of life, and the very skin that was made black, will by the same process of moisture and light, be rendered colourless. Whenever the vital principle exists in an active state, seclude the individual from light, and it loses part, or the whole of its colour; but dead matter is bleached by exposure to the action of that sun which would have deepened its hue had it possessed vitality. Matter possessing the vital principle, and exercising the functions of life, has its hues rendered deep, rich, or brilliant, by exposure to the sun's rays; but by the same process dead matter is gradually deprived of colour, and at length becomes white. This is too obvious to require illustration by particular instances; but the mode of action in the principle that influences these facts is not yet explained.

The natural history of man, as exemplified in the effects of habit and locality, is strongly characterized in the view of Cretinism by Dr. Reeve. In the wretched Cretin is seen, not only the human form altered and debased, more than in any other instance, but by external circumstances the mental principle entirely extinguished. Among the Alps of Savoy and Switzerland, there are vallies so deeply embosomed that the breeze is never felt in them; these vallies are exposed to the direct and still more to the reflected rays of the sun: in many of these the effluvia from marshes are very strong, and the atmosphere humid, close, and oppressive. It is among the poor inhabitants of these ill ventilated, hot, and humid vallies, that Cretinism is produced. All the Cretins that I saw, says Dr. Reeve, were in adjoining houses, in the little village *la Batia*, situated in the narrow corner of a valley; these houses being built up under ledges of rocks, and all of them very filthy, very close, and very hot.

The appearances that characterize Cretinism, are weakness ness of body, and imbecility of mind. "The enlargement of the thyroid gland, called *goitre,* though not a constant attendant, is a most unsightly feature in the aspect of a Cretin. His head is also deformed, his stature diminutive, his complexion sickly, his countenance vacant, his lips and eyelids coarse and prominent, his skin wrinkled and pendulous, his muscles loose and flabby. The qualities of the mind correspond to the deranged state of the body which it inhabits, and imbecility prevails in all the intermediate degrees, from excessive stupidity to complete fatuity."

To Dr. Reeve science is indebted for this addition to the history of a moral and physical degradation, to which human nature in no other form or circumstance of disease has been condemned. But this has yet left much for the investigation of philosophers, legislators, physicians, and naturalists. The melioration of the condition of man, is, or should be, the great object of intellectual exertion. In Cretinism we see the lowest point to which he can be reduced—weak in body, imbecile in mind. The natural and moral causes producing this have been often sought for, perhaps they have not yet been found. It is even uncertain when this peculiar state of humanity was first observed. As connected with bronchocele, and till very lately it was believed that these diseases had the closest affinity; and that the Cretin was always a goitre, was perhaps known to Pliny, Horace, and Juvenal; but the first writer who described it with the distinctness of natural history, or with the views of a physician, was *Felix Plater,* in his Praxeos Medica.* Since this, it has been frequently

* The *Prax. Med.* was first published at Basle, 3 vols. 1602; again, 3 vols. 8vo. 1625, and 1656. The last edit. 4to. 1736, with a Preface by Emanuel Koenig. Of those who have written on Cretinism, we are enabled to enumerate Prof. Akerman, Ueber die Cretenin, eine besondere Menschenabart in den Alpen, 8vo. Gotha, 1795.—Fodere, essai sur le Goitre et Cretinisme, Turin, 1794; Paris, 1800.—Coxe's Travels in Switzerland.—De Saussure, and Sir George Staunton.

It is evident that Cretinism is not confined to the Alpine vallies of Switzerland; it has been met with in Tartary; and in the Reserches Philosophiques sur les Americains, Cretins are compared with the Blafards of the Isthmus of Darien. An Englih physician, who travelled through Styria and Carinthia in 1706, describes the inhabitants of these places as very poor, especially in Styria; and great numbers of them *blind and foolish,* their skins of a pale yellow, with large scrophulous tumours filling up all the space of the throat from the chin and upper part of the breast, and weighing some pounds. I examined, says he, the persons and consulted their physicians, and they all agree that the whole of these tumours ly noticed by philosophers and physicians; and the last of them, Dr. Reeve, was led to the inquiry by the hope of discovering some function for the thyroid gland, more satisfactory than what is commonly alleged. But in this he was disappointed; for it appeared that the disease in the thyroid gland, called bronchocele, was not an essential part of Cretinism, for it often appeared without that disease of intellect, and the Cretin was frequently free from bronchocele.

The connection that chemistry holds with every branch of science and art, the explanations it has afforded, under the denomination of animal chemistry, of the constituent parts of animal bodies; and the elucidation that respiration, in particular, has received from it, combine to make its discoveries of high importance to the medical practitioner. In the history of the progress of this art, a very few years have sufficed to mark its pre-eminence in Germany and France; but the period, perhaps, has now arrived when Britain is destined to manifest a superiority: and in the brilliant discoveries of Prof. Davy, we hail the dawn of a reputation that will place us above the level of the chemists on the Continent. If the late discovery of the base of alkalies, made by this ingenious chemist, has not an obvious connection with the medical art, it is in itself so striking, affords such numerous analogies, and opens so many new views, that we feel fully justified in giving it a short detail.

In the year 1807 Mr. Davy proved, "in examining the agencies of galvanism in the decomposition of a variety of alkaline and earthy salts, that the formation of all chemical compounds depend on the electrical state of the mours are substantial flesh, that they are born so, and are incurable. From Cleopritz, at the foot of the mountains, in the space of 150 miles, I saw, he adds, some thousands of these miserable creatures. Though this is to be considered as an historical fact, and the first, perhaps, on Cretinism that appeared in English; yet we cannot subscribe to the opinion of this gentleman, that the cause of this disease was "drinking cold mineral waters on the Alps," notwithstanding he supports his hypothesis by large citations from *Baccius*, a physician of great celebrity at Rome in the 16th cent. who wrote with much learned research on a variety of subjects. Among his works is a folio de Thermis, Lacubus, Fluminibus Balneis totius orbis, which passed through many editions; to the last of these was added a chapter de nova methodo Thermarum explorandarum, deque minera et viribus Fontium Medicatorum. In the former part of this Note the Memoir of the Count de Maugiron expressly on Cretinism, read to the Roy. Soc. at Lyons about 30 years ago, was overlooked.

the materials of which they are composed." Curious and important as is this fact, it claims additional attention, as having led to the discovery of another of extraordinary brilliance, and deeply affecting many of the principles of natural science. Following on his researches into the chemical changes produced in substances of known composition by electricity, Mr. Davy concluded that this new method of investigation promised to lead to a more intimate knowledge than had hitherto been obtained, concerning the true elements of bodies. At length, he was able to state, "that in the course of a laborious experimental application of the powers of electro-chemical analysis, to bodies which have appeared simple when examined by common chemical agents, or which, at least, have never been decomposed, it was his good fortune to obtain new and singular results." These new and singular results were a complete decompositon of *potash* and *soda,* and a demonstration that their base is a shining metallic substance closely resembling quicksilver.

In the first attempts which Mr. Davy made to decompound the fixed alkalies, he failed, in consequence of having acted on their aqueous solutions only. But when a small piece of pure potash, moistened a little by the breath, was placed upon an insulated disc of platina, connected with the negative side of a galvanic battery, consisting of 100 plates of 6 inches, and 150 of 4 inches square, in a state of intense activity, and a platina wire, communicating with the positive side, was brought in contact with the upper surface of the alkali, a vivid action soon commenced. The potash began to fuse at both its points of electrization, *and small globules having a high metalic lustre, and precisely similar in visible characters to quicksilver, appeared*; some of which burst with explosion and bright flame. These globules are the basis of potash That alkali being composed of this peculiar base and oxygen only; soda, when acted upon in the same manner, exhibited an analagous result. Mr. Davy conceives the bases of the two fixed alkalies to be metals, and he has named one *Potasium,* and the other *Sodium.* The ingenious discoverer of these new metals, found that they could only be preserved and confined, so as to investigate their properties and submit them to experiments, by being immersed in recently distilled naphtha, and that when covered with a thin film of this fluid, their physical properties may be easily examined in the atmosphere.

The discovery of this property in naphtha, enabled Mr.

Davy

Davy to ascertain that when a globule of the base of potash was exposed to the atmosphere, it immediately attracted oxygen, and a white crust formed upon it, which proved to be pure potash. That when the globules were strongly heated, and then suspended in oxygen gas, a rapid combustion with a brilliant flame was produced, and these metallic globules were converted into an alkali, whose weight greatly exceeded that of the combustible matter consumed. That *Potasium,* at 60° Fahrenh. is only imperfectly fluid; at 70° it becomes more fluid; and at 100°, its fluidity is perfect, so that different globules may be made to run into one. At 50° it becomes a soft and malleable solid, which has the lustre of polished silver, and at about the freezing point of water it becomes harder and brittle, and when broken into fragments exhibits a crystallized texture, of perfect whiteness and high metallic splendour. To be converted into vapour, it requires a temperature approaching to that of red heat: it transmits caloric with great facility, and is a perfect conductor of electricity. Resembling the metals in all these properties, it is, however, remarkably different from any of them in specific gravity. It swims in distilled naphtha whose specific gravity is only .770, water being considered 1,000. Its specific gravity is to that of mercury as 10 to 223. When *Potasium* is introduced into oxymuriatic gas, it burns spontaneously with a bright and red light, and muriate of potash is formed. When thrown upon water, *Potasium* decomposes it with great violence, an instantaneous explosion is produced with brilliant flame, and a solution of pure potash is the result. When a globule is placed upon ice, not even the solid form of the two substances can prevent their union; for it instantly burns with a bright flame, and a deep hole is made in the ice, which is found to contain a solution of potash. Thrown into mineral acids it flames and burns on the surface. In sulphuric acid, sulphate of potash is formed; in nitrous acid, nitrous gas is disengaged, and nitrate of potash formed. When brought in contact with phosphorus, and pressed upon, there is a considerable action: they become fluid together, burn, and produce phosphate of potash. If a globule of *Potasium* is made to touch a globule of mercury about twice as large, they combine with considerable heat; the compound is fluid at the temperature of its formation; but when cool it appears a solid metal, similar in colour to silver. When a globule of this amalgam is thrown into water, it rapidly decomposes it with a hissing noise, potash potash is formed, hydrogen disengaged, and the mercury remains free. This basis of potash readily reduces metallic oxides when heated in contact with them; it decomposes common glass by a gentle heat, and at a red heat, effects a change even in the purest vitrefactions.

*Sodium*, or the base of soda, like that of potash, is white, opake, and has the lustre of silver. The property of welding, which belongs to iron and platina, at a white heat only, is possessed by this substance at common temperatures. In its more obvious properties it is very similar to the base of potash; but has a greater specific gravity, being to that of water nearly as 9 to 10, while *Potasium* is nearly as 6 to 10. In oxygen gas it produces a white flame, and sends forth bright sparks, occasioning a beautiful effect. In oxy-muriatic acid gas it burns vividly, with numerous scintillations of a bright red colour. In the quantity of $\frac{1}{40}$ it renders mercury a fixed solid, of the colour of silver, and forms an alloy with tin. When amalgamated with mercury, the amalgam will combine with other metals; and there was reason to believe that platina and iron remain in combination with the mercury, even when deprived of the new substance by exposure to the air.

For this short view of the history of a discovery which has attracted the attention of scientific persons in an extraordinary degree; and the concise statement of some of the physical properties of *Potasium and Sodium*, we are chiefly indebted to the ingenious Mr. Parke. Of the probable results from the discovery of these new substances, Mr. Davy himself is, perhaps, the most accurate judge. An immense variety of objects of research, he says, is presented in the powers and affinities of the new metals produced from the alkalies. In themselves, they will undoubtedly prove powerful agents for analysis; and having an affinity for oxygen stronger than any other known substances, they may possibly supersede the application of electricity to some of the undecompounded bodies. In sciences kindred to chemistry, the knowledge of the nature of the alkalies, and the analogies arising in consequence, will open many new views; they may lead to the solution of many problems in geology, and shew that agents may have operated in the formation of rocks and earths which have not hitherto been suspected to exist.

Among the multitude of objects which chemistry embraces, there is no one that should more interest the medical profession than the analysis of mineral waters. From the remotest records of time, mineral springs have been considered as natural remedies of great efficacy; and many efforts have been made to illustrate their operation by ascertaining their contents. How aukward have been the means, and how imperfect have been the results, need scarcely be mentioned, when it is known that till the present year, the waters of Cheltenham had not undergone an analysis that could be confided in. From an elaborate examination of the spring originally resorted to at this celebrated place, it is pronounced by Mr. Accum to contain in a gallon of water,

| | Grains |
|---|---|
| Muriate of soda | 219,75 |
| Sulphate of magnesia | 98,25 |
| ——— soda | 80,01 |
| Muriate of magnesia | 40,00 |
| ——— lime | 36,00 |
| Carbonate of iron | 7,15 |
| Sulphate of lime | 85,01 |
| | 566,17 |
| | cub. inches |
| Carbonic acid gas | 12,07 |
| Oxygen gas | 4,03 |
| Atmospheric air | 1,21 |
| | 17,31 |

An anylysis of five other springs that have been more recently discovered in the same place, has been conducted with equal skill and attention.

In the Report of last year it will be seen that considerable attention had been given to states of the atmosphere, as affecting the health of mankind, and comparisons were made between the atmospherical influences of former periods and the present time; as well as attempts to ascertain the degrees of change produced in the air, by processes going on upon the surface of the earth, and the modes and qualities of impregnations so received. If the opinions on these subjects were often problematical, and have since been frequently controverted, it was still satisfactory to see that the subject drew attention, and that this attention promised, by degrees, to clear away the obscurity that hung over it, and that a just sense was taking place of the value and importance of those investigations. Indeed, diseases of the most alarming kinds, contagions and epidemic epidemics of every denomination, either arise out of some peculiar state of the atmosphere, or depend on some properties in that fluid for their speedy or slow promulgation; so that every inquiry into its laws and history, though it may in some points be obscure, erroneous, or inartificially conducted, must be received by the thinking part of the profession, with an attention that is due to efforts employed on the elucidation of this difficult but important subject. With these sentiments it cannot but be acknowledged, that "a general view of the natural history of the atmosphere, and of its connection with the sciences of medicine and agriculture," compiled with a view to bring into a moderate compass, the whole of the facts and discoveries on this subject, with observations on the causes of epidemics, excited considerable expectations combined with the fullest approbation of the design. But if on a subject of extreme intricacy, embarrassed with a multitude of obscure and uncertain bearings, disturbed by contending opinions, and involved in the approximations and repulsions of old and new theories, the execution should not have fully risen to the point of expectation; still much information has been collected, and many difficulties have been subdued.

There is, however, yet wanted on the natural history of the atmosphere, a work of moderate price, which should clearly state all the known and admitted facts,—shortly, but explicitly giving the history of their discovery, the experiments and the arguments by which they are supported, as well as the objections brought against them;—their effects in arts and sciences, and their particular and general influence on the operations of Nature. Extreme care should be taken that no extraneous matter be admitted into a manual or catechism of this kind, that the relations be concise but perspicuous, that the experiments be so detailed as to admit of being repeated, and that the references be so distinct as to allow of an easy application to the original sources of information. To practitioners in medicine, to gentlemen, and to intelligent farmers, such a manual would probably be very acceptable.

(To be continued in our next, at p. 105.)

